# A liquid biopsy for bronchopulmonary/lung carcinoid diagnosis

**DOI:** 10.18632/oncotarget.23820

**Published:** 2017-12-29

**Authors:** Mark Kidd, Irvin M. Modlin, Ignat Drozdov, Harry Aslanian, Lisa Bodei, Somer Matar, Kyung-Min Chung

**Affiliations:** ^1^ Wren Laboratories, Brandford, CT, USA; ^2^ Yale University School of Medicine, New Haven, CT, USA; ^3^ Memorial Sloan Kettering Cancer Center, New York, NY, USA

**Keywords:** biomarker, bronchopulmonary, carcinoid, liquid biopsy, lung

## Abstract

No effective blood biomarker exists to detect and clinically manage bronchopulmonary (BP) neuroendocrine tumors (NET). We developed a blood-based 51 NET-specific transcript set for diagnosis and monitoring and evaluated clinical performance metrics. It accurately diagnosed the tumor and differentiated stable from progressive disease as determined by RECIST criteria. Gene expression was evaluated in: a) publicly available BPNET transcriptomes (GSE35679); b) two BPNET cell-lines; and c) BPNET tissue with paired blood (*n* = 7). Blood gene expression was assessed in 194 samples including controls, benign lung diseases, malignant lung diseases and small bowel NETs. A separate validation study in 25 age- and gender-matched BPNETs/controls was performed. Gene expression measured by real-time PCR was scored (0–100%; normal: < 14%). Regression analyses, Principal Component Analysis (PCA), hierarchical clustering, Fisher's and non-parametric evaluations were undertaken. All 51 genes were identified in BPNET transcriptomes, tumor samples and cell-lines. Significant correlations were evident between paired tumor and blood (R2:0.63–0.91, *p* < 0.001). PCA and hierarchical clustering identified blood gene expression was significantly different between lung cancers and benign diseases, including BPNETs. Gene expression was highly correlated (R^2^: 0.91, *p* = 1.7 × 10-^15^) between small bowel and BPNET. For validation, all 25 BPNETs were positive compared to 20% controls (*p* < 0.0001). Scores were significantly elevated (*p* < 0.0001) in BPNETs (57 ± 28%) compared to controls (4 ± 5%). BPNETs with progressive disease (85 ± 11%) exhibited higher scores than stable disease (32 ± 7%, *p* < 0.0001). Blood measurements accurately diagnosed bronchopulmonary carcinoids, distinguishing stable from progressive disease. This marker panel will have clinical utility as a diagnostic liquid biopsy able to define disease activity and progression in real-time.

## INTRODUCTION

Bronchopulmonary neuroendocrine tumors (BPNETs) or “carcinoids” comprise a spectrum of tumors that arise from respiratory neuroendocrine cells. They represent ~ 25% of lung neoplasia and ~ 30% of neuroendocrine tumors (NETs) [1]. No effective diagnostic biomarker in blood is available and imaging cannot specifically identify a BPNET [2]. Chromogranin A (CgA) is effective in BPNET tissue as an identifier of NET but its measurement in blood has limited clinical utility [3]. Hence, there is no real-time method to monitor disease treatment or progress [4]. Imaging is relatively insensitive and suboptimal for early diagnosis, while disease monitoring is expensive and has radiation exposure concerns [5]. The absence of a blood biomarker or liquid biopsy for BPNETs is thus a critical unmet need.

Lung carcinoid tumors are histologically differentiated into “typical” and “atypical” phenotypes. This subtyping has been related to the Ki67 labeling index; typical carcinoids (TC) are associated with a Ki67 < 5% and atypical carcinoids (AC) < 20% [6]. The Ki67-based proliferation index, however, does not effectively differentiate TC and AC although it may provide prognostic information [7]. Tumor heterogeneity and the invasiveness of biopsy significantly limits repeated tissue-based evaluations [8, 9].

Precise clinical evaluation is necessary since 50% of ACs develop metastasis in < 2 years of diagnosis and 15% of TCs metastasize within 4 years [10]. Moreover, post-resection recurrences occur in 5% of TC and 20% of AC [11]. However, neither standard histology nor Ki67 counting accurately predict tumor behavior. Long-term surveillance is therefore recommended [10, 12], but strategies available to undertake this are limited.

Tissue transcriptome evaluations have provided information about the etiopathogenesis and molecular classification of lung carcinoids [13, 14]. Potential diagnostic [15] and prognostic markers [16] have been identified. The clinical value of these tissue-based approaches including expression of CD44, the orthopedia homeobox (*OTP*) gene, stathmin or desmoglein 3, however, remain to be determined [16]. Given the invasive nature and the technical limitations of tissue biopsy, there is enthusiasm for the development of surrogate markers that can be quantified in blood on a real-time basis [17]. Such “liquid biopsies” have been effective in lung neoplasia e.g., for monitoring treatment responses to EGFR inhibitors through identification of mutation T790M in EGFR in circulating tumor DNA [18, 19]. The goal of developing similar biomarker tools is a critical unmet need in bronchopulmonary neuroendocrine neoplasia [2].

The measurement of molecular signals such as circulating tumor DNA, methylated gene targets or circulating tumor cells have been limited in NETs [20]. Their application has not been considered in BPNETs [21]. However, recent reports that neuroendocrine tumor-associated mRNAs are detectable and quantifiable in blood has raised the consideration that this strategy might be effective in BPNETs [2]. Multianalyte gene measurement in blood of gut neuroendocrine tumors has been demonstrated to provide information of clinical utility [22, 23]. A 51-gene expression test for gastro-entero-pancreatic (GEP) NETs has been developed and validated [17, 24]. The score expressed can identify tumor presence or absence, identify residual disease and recurrence, distinguish stable from progressive disease and identify the efficacy of treatment [22–24]. Omic analysis of tumor transcriptomes can be interrogated to identify candidate gene targets and provide predictive information relevant to the biological behavior of an individual tumor [17].

GEP-NETs share many similarities with BPNETs. Both types of tumor arise from the diffuse neuroendocrine cell system, secrete bioactive peptides and amines, and consequently exhibit similar functional symptomatology (bronchospasm, flushing, diarrhea). Based on this broad commonality of cellular origin and secretory pattern, we hypothesized that BPNETs would express similar transcripts to GEP-NETs and that these could be identified in circulating blood.

Our aims therefore were as follows. Firstly, to examine whether a GEP-NET derived 51 neuroendocrine neoplastic marker gene signature was detectable in BPNET transcriptomes. Secondly, to evaluate whether gene expression was measurable in lung tumor tissue and in human lung neuroendocrine tumor cell lines. Thirdly, to determine whether blood transcript levels correlated with the tumor tissue obtained from the same patient. Finally, to assess clinical applicability we confirmed that the 51-marker gene expression was present in blood in pilot and validation cohorts and that gene expression levels could differentiate progressive from stable disease.

## RESULTS

### BPNET transcriptomes–51-marker gene signature evaluation

**Figure 1:**
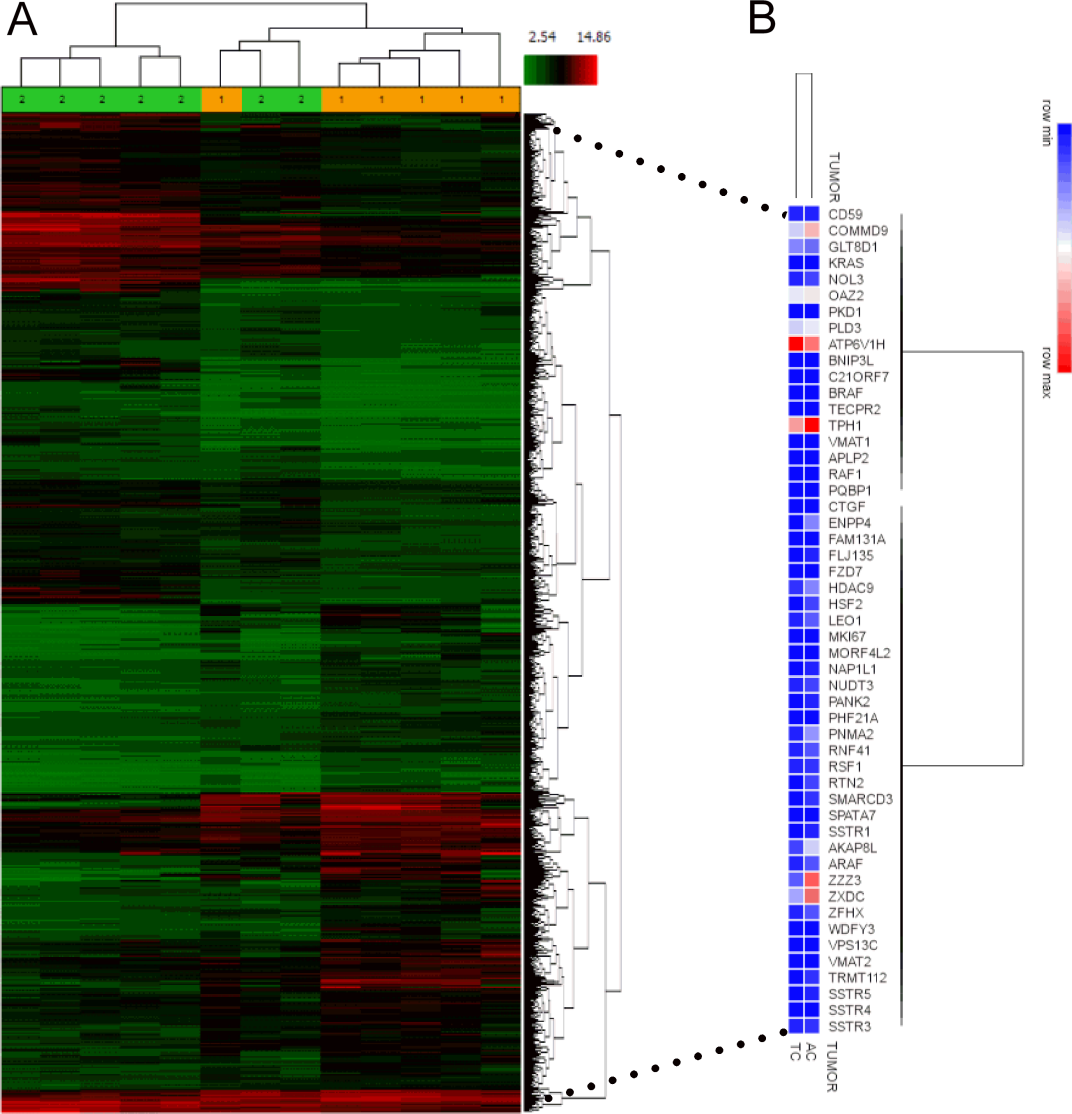
NETest gene expression in BPNET transcriptomes (**A**) Analysis of GSE35679 identified that histologically different lung carcinoid tumors could not be separated by heirarchical clustering. Subtypes were not homogeneous at a global transcriptome level. 1 = TC; 2 = AC. (**B**) Evaluation of the expression (averaged in each of the two tumor subtypes) of each of the 51 NETest genes identified substantial, overlapping expression in both TC and AC. Of the 51 genes measured, 48 (94%) were similarly expressed.

The publicly available BPNET transcriptome array (GSE35679), which comprises 6 TC and 7 AC transcriptomes [15], was evaluated. Hierarchical clustering of global gene expression identified that these tumor subtypes could not be separated at a transcript level (Figure 1A). This identifies that lung carcinoids, irrespective of histological classification, express common genes.

We then determined whether a 51 GEP-NET derived marker gene signature from gut NETs was detectable in the public transcriptomes. All tumors–whether TC or AC–expressed all genes. Log_2_ bi-weighted expression levels (Table 1) ranged from 3.33 (*MKi67*) to 12.57 (*ARHGEF4* - Rho guanine nucleotide exchange factor 4/FLJ135) in TC and 3.41 (*MKi67*) to 11.81 (*SSTR5–*somatostatin receptor 5) in AC. Mean levels of the 51 marker genes were 7.71 (TC) and 7.98 (AC). We identified a house-keeping gene *ALG9* (alpha-1,2-mannosyltransferase) [25] used to normalize gene expression from NET tumor and blood, in all samples. In the two histological subtypes, the average log_2_ bi-weighted expression for *ALG9* ranged from 7.96–8.36.

**Table 1:** Gene expression in transcriptomes and in model lung NET cell lines

Gene	Log^2^ bi-weighted average signal (Transcriptomes)		Normalized Gene Expression^*^(Cell Lines)	
	AC	TC	H720 AC “like”	H727 TC “like”
*ALG9*	8.36	7.96	-	-
*AKAP8L*	10.47	9.78	0.38	0.003
*APLP2*	6.54	6.61	0.29	0.01
*ARAF1*	9.18	9.08	0.23	9 × 10^−5^
*FLJ10357 (ARHGEF40)*	11.42	12.57	0.007	0.0001
*ATP6V1H*	4.7	5.18	1.72	0.007
*BNIP3L*	7.1	7.1	0.003	0.0017
*BRAF*	5.3	5.38	3.28	0.009
*C21ORF7 (MAP3K7 C-terminal like)*	9.42	8.6	0.37	0.008
*CD59*	8.13	8.21	0.46	0.01
*COMMD9*	11.16	11.22	0.17	0.0002
*CTGF*	7.0	6.93	0	0.12
*ENPP4*	9.74	7.6	1.21	0.006
*FAM131A*	4.59	4.45	2.30	0.0006
*FZD7*	7.5	7.04	0.04	0.004
*GLT8D1*	4.3	3.91	2.36	0.015
*HDAC9*	9.67	10.58	0.15	0.001
*HSF2*	9.83	9.44	0.55	0.002
*KRAS*	8.92	7.9	1.17	0.016
*LEO1*	5.29	5.33	2.69	0.09
*MKi-67*	3.41	3.33	1.04	0.38
*MORF4L2*	6.86	6.73	0.13	0.005
*NAP1L1*	7.6	6.95	0.17	0.16
*NOL3*	8.87	8.98	0.09	0.0003
*NUDT3*	9.02	8.93	0.06	0.0004
*OAZ2*	10.97	11.44	0.29	0.002
*PANK2*	7.98	6.93	0.064	0.0001
*PHF21A*	6.39	5.28	0.02	0.0005
*PKD1*	4.81	4.95	1.07	0.001
*PLD3*	10.71	11.3	0.88	0.0009
*PNMA2*	10.08	9.06	0.63	0.014
*PQBP1*	7.4	7.45	0.42	0.15
*RAF1*	6.68	6.9	0.12	0.0007
*RNF41*	9.23	9.02	0.12	0.01
*RSF1*	8.54	8.51	1.32	0.28
*RTN2*	8.99	8.02	0.44	0.0009
*SMARCD3*	8.21	7.38	0.35	0.003
*SPATA7*	8.83	8.59	4.61	0.30
*SSTR1*	5.3	4.86	0	5.99
*SSTR3*	8.38	7.71	0.02	0.001
*SSTR4*	6.88	7.07	0.006	0.002
*SSTR5*	11.81	12.01	0.018	0.09
*TECPR2*	8.63	7.1	0.05	0.0001
*TPH1*	6.22	6.77	0.67	0.007
*TRMT112*	5.49	4.91	0.34	0.005
*VMAT1 (SLC18A1)*	8.52	7.9	0	0.003
*VMAT2 (SLC18A2)*	3.91	3.69	11.47	0.03
*VPS13C*	5.93	5.49	0.1	0.04
*WDFY3*	5.48	5.1	0.04	6×10-5
*ZFHX3*	9.22	8.95	0.85	0.005
*ZXDC*	11.47	10.94	0.11	0.002
*ZZZ3*	11.56	10.14	1.22	0.02

^*^Target genes normalized to *ALG9* [25].

Hierarchical clustering of the averaged 51 marker gene expression from 6 TC and 7 AC transcriptomes identified that the averaged expression of 48 of the 51 genes (94%) overlapped (Figure 1B). AC tumors typically expressed 2-3-fold higher levels of *ZZZ3* (ZZ-type zinc finger-containing protein 3) and *ENPP4* (ectonucleotide Pyrophosphatase/Phosphodiesterase 4) with low ( < 0.4-fold expression) of *ARHGEF4* compared to TC. Overall, all 51 marker genes were commonly expressed in both BPNET histological subtypes. We therefore evaluated the expression levels as biomarkers.

### BPNET cell line confirmation of gene expression

**Figure 2:**
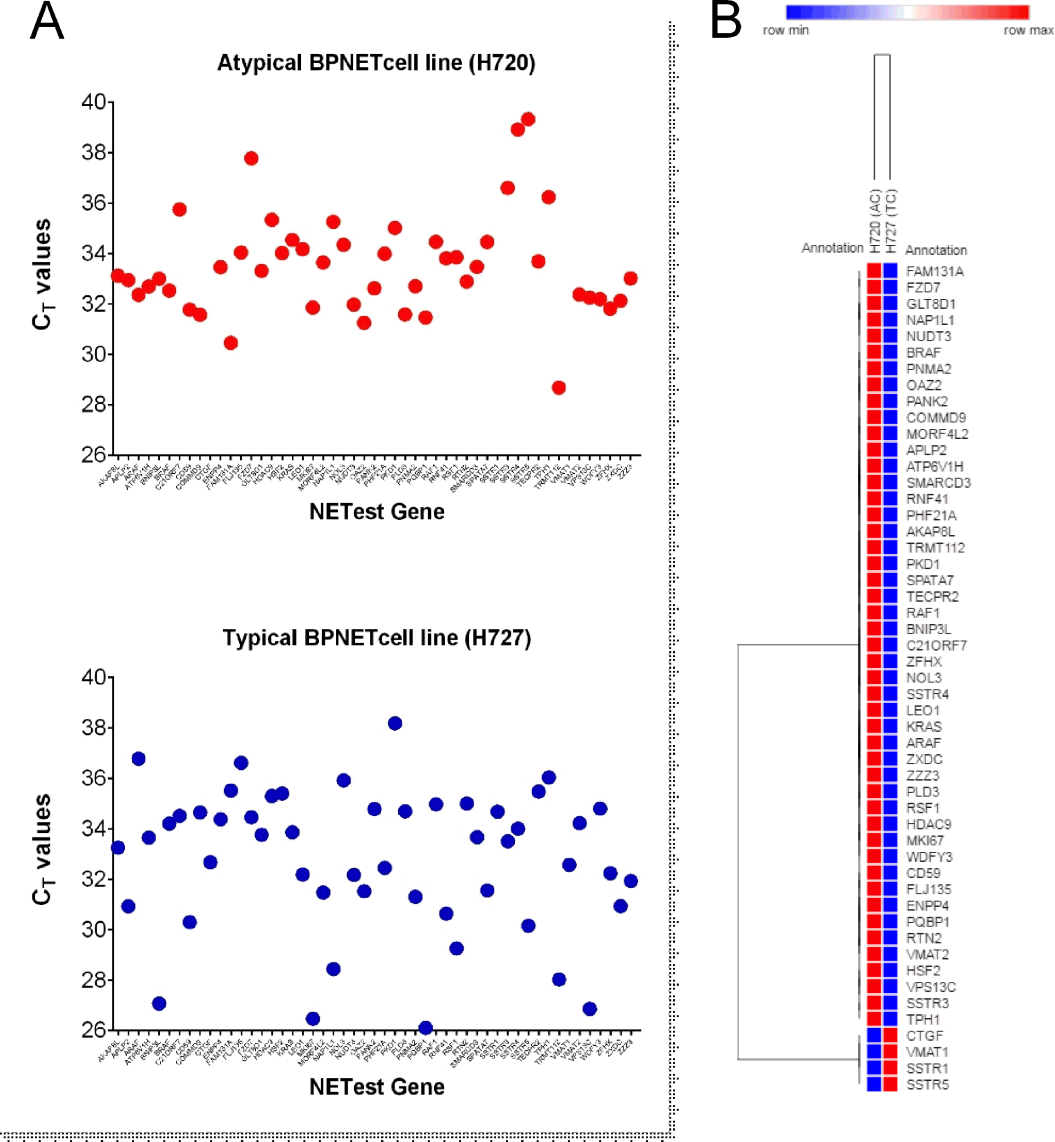
PCR analysis of the 51 marker genes in two lung NCI-NET cell lines, H720 (AC-like) and H727 (TC-like) (**A**) All 51 genes (100%) were amplifiable (CT<40 cycles) in H727 and 48 (94%) were detected in H720. (**B**) The more aggressive AC cell line (H720) exhibited higher expression in 92% of the genes; H727 was associated with higher expression of genes involved in fibrosis (CTGF), amine secretion (VMAT1) and somatostatin receptors (SSTR1, 5).

Real-time PCR of mRNA isolated from two model lung neuroendocrine cell lines that represent each carcinoid histological subtype (H720 and H727) identified that 48 (94%) of the marker genes were expressed in H720 while 100% were detectable in H727. Cycle times (C_T_) ranged from 26 to 39 (Figure 2A). The AC-like cell line (H720) expressed elevated expression of 47 (92%) of the 48 genes detectable (Table 1, Figure 2B) compared to the TC-like cell line. In the TC subtype (H727) *CTGF* (connective tissue growth factor), *SSTR1* (somatostatin receptor subtype 1) and *VMAT1* (*SLC18A1*) were also detected. *SSTR5* was also significantly over-expressed in this subtype. These data identify that the marker genes are transcribed and are detectable in cell lines derived from lung bronchopulmonary carcinoids. The subtle differences in expression noted may represent the divergent biological behavior of TC/AC tumors.

### Confirmation in matched blood and tumor tissue of gene expression quantification

**Figure 3:**
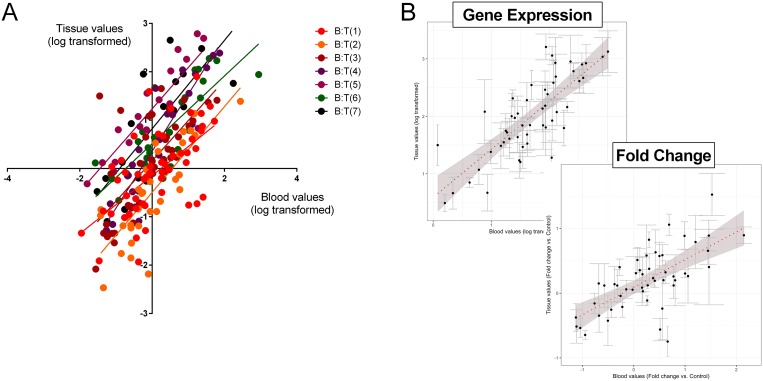
Correlation between the 51 marker genes in matched tumor tissue and circulating blood (**A**) Linear regression (Spearman) analysis of log transformed normalized values of each of the individual tumor-blood pairs identified R2 to range from 0.63 (T1:B1; *p* < 0.001) to 0.91 (T4:B4; *p* < 0.001). T = tumor; B = matching blood. 3B. Normalized gene expression in the grouped samples by linear regression (Pearson and Spearman) analysis of log transformed normalized values identified the R2 to be 0.79 (*p* = 3.3 × 10–12) and the Spearman Rho to be 0.77 (*p* = 2.2 × 10–16). For evaluation of the relationship between gene expression (expressed as a fold-change versus normal tissue or blood) identified the R2 to be 0.67 (*p* = 6.6 × 10–8) and the Spearman Rho to be 0.64 (*p* = 7.4 × 10–7). In both graph plots (**B**), the 7 pairs (blood-tissue) were averaged and error bars indicate standard error of the mean. The dotted red line is the best linear fit line to the dataset. Shaded area reflects standard error of the fit.

We next evaluated gene expression in matched tumor tissue and blood sample pairs (*n* = 7). All samples expressed detectable mRNA of all 51 marker genes irrespective of the histological subtype (TC: *n* = 2 or AC: *n* = 5) or source (tissue or blood). Averaged normalized gene expression levels in tumor tissue ranged from 0.03 (*PHF21A*–PHD Finger Protein 21A) to 161.0 (*CTGF*) and in blood from 0.01 (*PHF21A*) to 329.0 (*CTGF*). The normalized gene expression in each of the individual tumor-blood pairs identified the Spearman correlation (R^2^) to range from 0.63 (Tumor pair 1) to 0.91 (Tumor pair 4) (Figure 3A). Expression of the 51 genes in all samples (as a group) demonstrated significant correlations between tumor and blood (Pearson: R^2^: 0.79, *p* = 3.3 × 10^−12^; Spearman: rho = 0.77, *p* = 2.2 × 10^−16^) (Figure 3B-top).

In tumor tissue, the averaged fold-change (tumor tissue compared to normal lung parenchyma) ranged from 0.92 (*PHF21A*) to 8.7 (*CTGF*). In blood, the averaged fold-change compared to expression in normal blood ranged from 0.01 (*PHF21A*) to 401.0 (*CTGF*). A comparison of the fold-change in tissue versus blood in all samples identified that the significant correlation for individual normalized gene expression (Figure 3A) was preserved (Pearson: R^2^: 0.67, *p* = 6.6 × 10^−8^; Spearman: rho = 0.64, *p* = 7.4 × 10^−7^) (Figure 3B-bottom).

These data demonstrate that gene expression in tumor tissue, irrespective of histology, is recapitulated in time-matched blood samples. Moreover, fold-changes in expression between tumor and normal are conserved irrespective of the source (tumor tissue or blood). Measurements of gene expression in blood therefore provide accurate and correlatable measurements of tumor tissue expression.

### Evaluation of gene expression in blood and validation of the signature as a circulating biomarker

**Figure 4:**
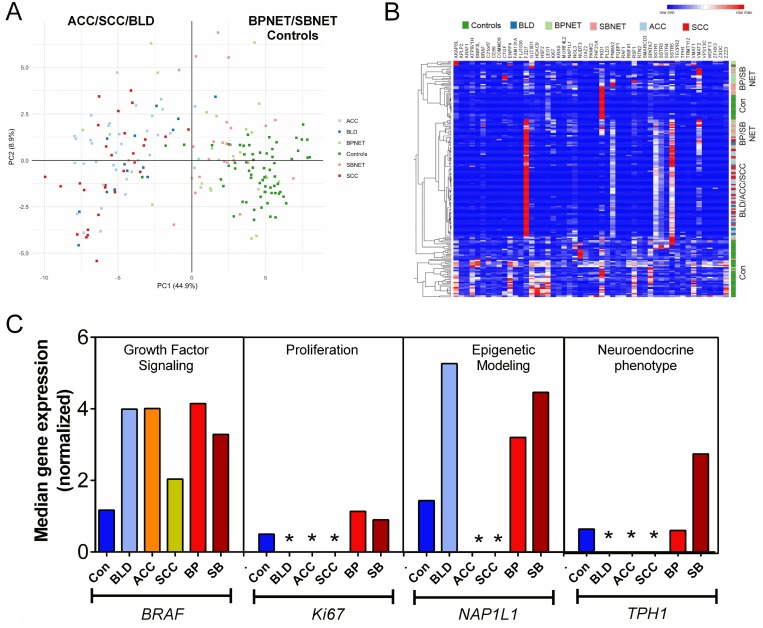
Evaluation of gene expression signature in pilot blood cohorts (**A**) PCA analysis of gene expression in whole blood from controls, benign lung disease, neoplastic lung diseases, BPNETs and small bowel NETs. The scatter plot visualizes the first and second Principal Components and respective variance percentages on the x- and y-axis respectively. Normalized expression of all 51 marker genes was used to reduce dimensionality. Each point represents a blood sample and distances between points correspond to similarities in gene expression, such that samples with similar gene expression profiles are placed closer together in the Principal Component Space. Gene expression of adenocarcinoma (ACC: *n* = 36) and squamous cell carcinoma (*n* = 29) as well as benign lung diseases (BLD: *n* = 14) were identified to be completely separated from controls (*n* = 65), BPNETs (*n* = 25) and SBNETs (*n* = 25). (**B**) Heirarchical clustering of gene expression identified that BPNET and SBNET clustered together as did ACC/SCC and BLD. Controls were separately grouped. (**C**) Canonical gene expression in controls, BLD, ACC, SCC, BPNETs and SBNETs. Growth factor signaling gene expression could be identified in all samples. Levels of BRAF were significantly elevated in BLD, ACC, BPNET and SBNET but were not elevated in SCC versus controls. Ki67 was rarely identified in BLD, ACC and SCC. When present, its levels were significantly lower in benign lung disease and lung cancers compared to BP- and SBNETs. NAP1L1, a marker of epigenetic remodeling and TPH1, a marker of neuroendocrine differentiation, were only expressed in controls, BPNET and SBNET. They were not expressed in BLD, ACC or SCC. ^*^p < 0.05 vs. Controls, BPNET and SBNET.

To confirm the observation that the target gene transcripts were detectable in blood, expression was evaluated in two separate cohorts. In the pilot cohort, normalized gene expression was evaluated in 194 samples including controls (*n* = 65), benign lung diseases (BLD: *n* = 14), BPNET (*n* = 25), small bowel (SB) NET (*n* = 25) and lung cancers: adenocarcinomas (*n* = 36) and squamous cell carcinomas (*n* = 29). A PCA analysis identified that the lung neoplasia and benign lung diseases were completely separated from the controls, BPNETs and SBNETs (Figure 4A). Hierarchical clustering determined a segregation between ACC, SCC and BLD and all other samples. BPNET and SBNET clustered together while controls were separate (Figure 4B). Analysis of neuroendocrine tumor pathways in BLD, ACC and SCC compared to controls, BPNET and SBNET identified significant differences in median expression. In particular, while *BRAF* was detected in all samples, *Ki67* was significantly lower in benign and malignant lung diseases than in controls and NETs (Figure 4C). Likewise, canonical markers for NET like *NAP1L1* and *TPH1* were also not identified in BLD, ACC and SCC. These data demonstrate that the markers detected are specific to and selectively define the biological nature of neuroendocrine tumors.

**Figure 5:**
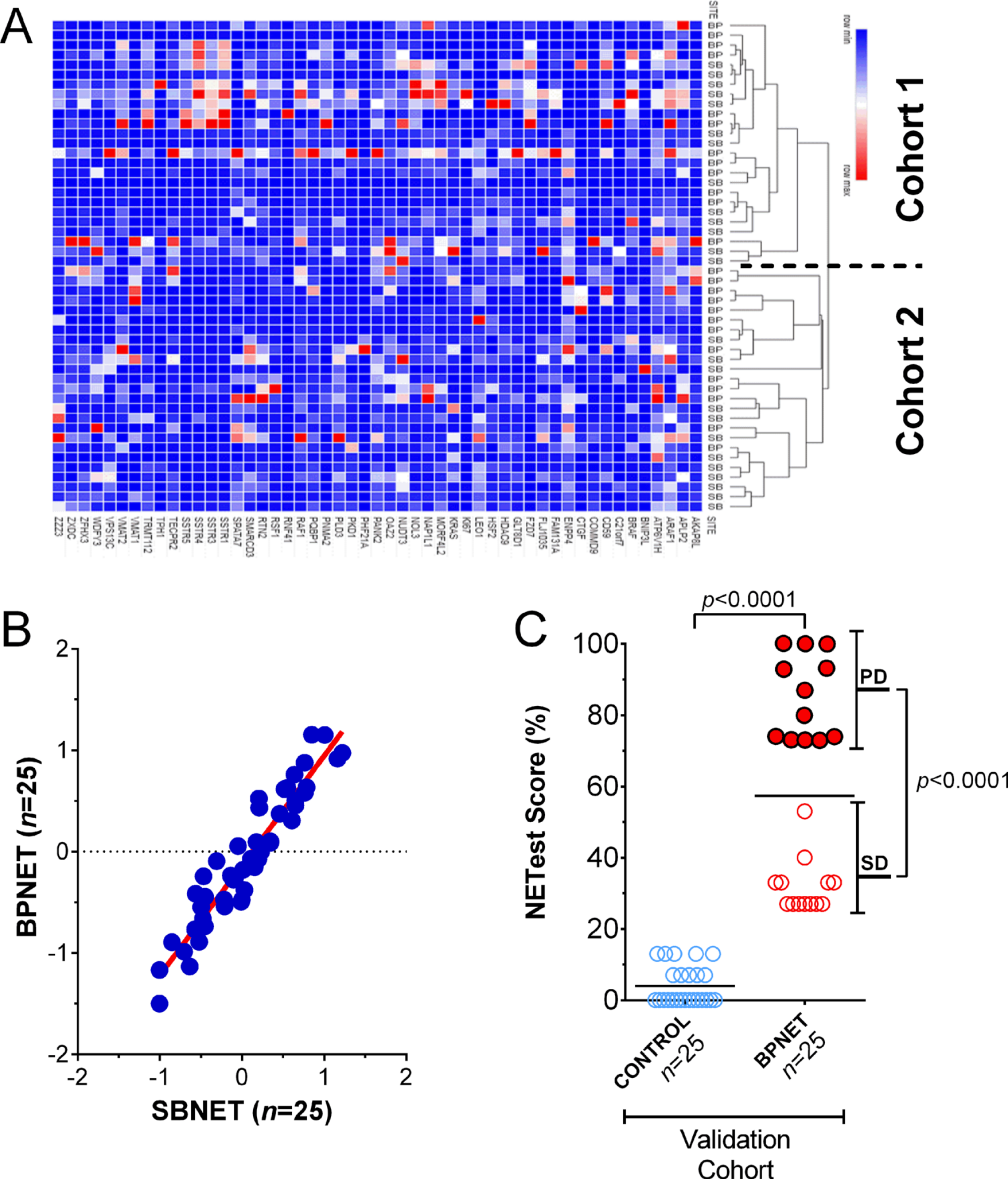
Confirmation of signature in BPNET blood (**A**) Gene expression was indistinguishable between BPNETs (*n* = 25) and SBNETs (*n* = 25). BPNETs comprised 48% of cluster cohort 1 and 52% of cohort 2 (*p* = 1.0). (**B**) Linear regression (Pearson and Spearman) analysis of log transformed median normalized gene expression values in BPNETs and SBNETs identified the R2 to be 0.95 (*p* = 1.1 × 10–17) and the Spearman Rho to be 0.91 (*p* = 3.6 × 10–15). (**C**) Expression represented as a score (0–100%) in age- and gender-matched BPNETs and controls. Levels were significantly elevated (*p* < 0.0001) in BPNETs compared to controls. A sub-analysis identified expression levels were significantly increased in BPNET with progressive disease compared to those with stable disease (*p* < 0.0001). Horizontal lines are the mean. ACC = adenocarcinoma, BP = bronchopulmonary, COPD = chronic obstructive lung diseases, SB = small bowel, SCC = squamous cell carcinoma. PD = progressive disease; SD = stable disease.

Further examination of the 25 BPNET and 25 SBNET blood samples using hierarchical clustering identified that gene expression was indistinguishable (Figure 5A). The two clustered cohorts comprised similar ratios of BPNET-SBNET samples–cohort 1: 12:13; cohort 2: 13:12 (*p* = 1.0). Additional evaluation of median expression of the 51 genes in all samples (when grouped) identified significant correlations between BPNETs and SBNETs (Pearson: R^2^: 0.95, *p* = 1.1×10^−17^; Spearman: rho=0.91, *p* = 3.6 × 10^−15^) (Figure 5B). In comparison, correlations (Pearson) for BPNETs and controls/BLD ranged from: 0.16-0.36 and for BPNETs and ACC/SCC were (0.44-0.46).

In the validation cohort of BPNETs (*n* = 25), normalized gene expression, expressed as the NETest score, was detected in all bloods (score: 57 ± 28%). In age- and gender-matched controls, low but positive expression levels were identifiable in 5 (20%; NETest score: 4 ± 5%). Scores were significantly elevated in the BPNET cohort (*p* < 0.0001) and significantly more samples were positive (100% vs. 20%; Fisher's exact test: *p* < 0.0001) (Figure 5C). A sub-analysis of the BPNETs identified no significant differences (*p* = 0.27) in expression levels between TC (NETest score: 43 ± 6%) and AC (NETest score: 60 ± 9%). In tumors that exhibited progressive disease, levels were higher (NETest score: 85 ± 11%, *p* < 0.0001) than stable disease (NETest score: 32 ± 7%).

These data therefore identify that the neuroendocrine specific multianalyte gene signature that identified GEP-NETs [24, 26] functions accurately as a biomarker in BPNETs.

## DISCUSSION

We have identified that 51 neuroendocrine tumor-related genes identifiable in small bowel NETs [24, 26], are present in bronchopulmonary NET (carcinoid) transcriptomes. Expression levels of all 51 target genes were detectable (average log_2_ bi-weighted signal ~ 8, on the arrays). This observation was confirmed by real-time PCR which identified expression of all 51 marker genes in mRNA isolated from seven matching clinical lung carcinoid samples. Tissue values correlated significantly with blood levels (R^2^ ~ 0.85). Two cell lines commonly used to model BPNET behavior [27, 28] also expressed the majority (94–100%) of the 51 marker genes. Gene expression in BPNET blood samples did not correlate with benign lung diseases and lung cancers.

Significant correlations were noted (R^2^ = 0.95) between gene expression in BPNETs and SBNETs. In BPNETs, we noted a significant correlation between blood marker expression and tumor tissue transcript levels similar to that observed in GEP-NETs. We therefore concluded that blood bioassay compartment accurately reflected BPNET tumor tissue-associated gene activity. In the second validation cohort, gene expression was confirmed to be quantifiable in BPNET blood. Levels were significantly elevated compared to age- and gender-matched controls and particularly increased in those with progressive disease compared to stable. These data identify that a 51 marker gene signature originally developed for GEP-NETs [17, 29] was applicable as a quantitative blood-based biomarker for BPNETs and could provide specific information relevant to disease status.

We examined the GSE35679 transcriptome to evaluate whether expression of the 51 neuroendocrine target genes were detectable at a transcriptome level. These arrays were originally developed and assessed by Toffaloria *et al.* [15] with the purpose of defining a gene expression signature to differentiate AC from TC at a tissue level. They identified 273 genes that were selectively upregulated in AC and focused on two gene products (GC-globulin or DBP: vitamin D-binding protein) and CEACAM1 (carcinoembryonic antigen family member) as potential biomarkers [15]. The authors proposed establishing immunohistochemical assays for the two candidates that could be used for routine cytological and histochemical diagnostic procedures [15].

Secretion of these markers into the circulation may provide an alternate source to evaluate. CEACAM1 is detectable in serum [30], and it has been used as a marker for pericarditis [31]. However, CEACAM1 is widely expressed in melanoma [32], non-small cell lung cancers [33] and lung adenocarcinomas [34], suggesting a degree of non-specificity as a biomarker. The vitamin D pathway plays a widespread role in different pathologies and exhibits extensive variations in blood levels [35]. It may therefore be difficult to establish GC-globulin as a clinically applicable circulating biomarker.

In our investigation of the same TC/AC transcriptomes, we noted that at a global transcriptomic level all tumors exhibited overlapping mRNA expression. While specific gene targets [15] may associate with histology, each of the two tumor subtypes expressed all 51 neuroendocrine tumor-associated genes. Although there is minor variance; namely *ZZZ3* and *ENPP4* are elevated in AC while *ARHGEF4* was over-expressed in TC, all 51 genes are expressed. All 51 can therefore be used as potential biomarkers for lung carcinoids.

The 51 gene transcripts are variously involved in regulating NET pathobiology. They included proliferation (*Ki67, NAP1L1, NOL3, TECPR2*), growth factor signaling (*ARAF, BRAF, KRAS, RAF1*), secretion (*TPH1, VMAT1*), epigenetic remodeling (*NAP1L1, RNF41, RSF1*) and somatostatin receptor expression (*SSTR1, 3, 4 and 5*). Ki-67 is well-described in BPNET [7], as is growth factor expression and signaling [36], the synthesis and transport of serotonin [36], epigenetic regulation [37] and somatostatin receptor expression [38]. It has been previously suggested that circulating levels of *SSTR5* could be a potential biomarker for lung NETs [39]. It is possible that some of these marker genes i.e., those involved in regulating proliferation or in regulating growth factor signaling may be detected in other lung diseases, especially cancers [40, 41]. While expression of *Ki67* and *BRAF* could be detected in ACC and SCC, levels were the same or significantly lower than controls. Other NETest markers involved in growth factor signaling and genes involved in the regulation of the neuroendocrine phenotype e.g., cell secretion and granule transport, or other canonical neuroendocrine marker genes, e.g., *TPH1* and *NAP1L1* [42, 43] were also scarcely detectable in lung cancers. Overall, our identification of the 51 marker neuroendocrine gene group in the lung carcinoid transcriptomes and the biological information regarding their functional roles provides the basis for their use as biomarkers to assess BPNETs.

The correlation between tumor and blood levels of the 7 matched tissue-blood sample pairs collected at surgery confirmed parallel expression of these genes in tissue and blood compartments. Blood measurements can function as surrogate markers of tumor tissue expression i.e., as a “liquid biopsy”, if levels are highly correlatable. In BPNETs we demonstrated a significant correlation (R^2^: 0.63–0.91, *p* < 0.001) between blood marker expression and transcript levels in tumor. Thus, blood is an appropriate compartment for assaying BPNET tumor tissue-related gene activity. In this respect, we previously demonstrated a similar relationship for GEP-NET tumor-blood pairs [17]. It seems likely that such a measurement will provide the basis for evaluating the 51 marker genes as a liquid biopsy for BPNETs.

In our test set of blood from BPNET compared to SBNET, all genes were detectable in blood samples of BPNET patients. Moreover, there was a significant correlation (R^2^ = 0.95) in expression of each of the 51 marker genes between these two tumor types. In the same set, gene expression in controls, benign lung disease and lung cancers were significantly different. Specificity compared to adenocarcinoma, squamous cell carcinoma, COPD and controls was demonstrated through PCA and hierarchical clustering. This demonstrated that the gene signature in BP- and SBNETs was significantly concordant. Correlation analysis of this data confirmed the poor correlation between BPNETs and other lung diseases (R^2^ = 0.16–0.46).

Given our demonstration of the significant association in gene expression between BPNET and SBNET, we therefore used the same mathematical approach that we developed for GEP-NETs to score gene expression in BPNET blood. For GEP-NETs, individual gene expression of the 51 markers is analyzed using four different mathematical tools: *Support Vector Machine*, *Linear Discriminant Analysis*, *K-Nearest Neighbors*, and the *Bayes Algorithm.* This categorization results in a 0-8 score [24, 44]. This naïve expression score is then converted to a clinical activity score ranging from 0% (low activity) to 100% (high activity) based on altered expression levels of genes mathematically determined to differentiate progressive from stable disease [17]. Elevated expression of these progression-associated genes is used to weight the score such that a high score e.g., “8” is scaled to 100% (high activity). A score of “8” with a low expression of “biologically aggressive genes” is weighted to 53%. We have previously determined the ranges that conform to clinical disease assessment in GEP-NETs: minimal activity: < 0–14%, low activity: 14-40%, and intermediate-high activity: > 40–100% [17]. We used the same basic approach in BPNETs and expect that some degree of modification of ranges will occur as larger cohorts of BPNETs are studied.

The results of this gene expression scoring in the independent age- and gender-matched BPNET/control validation cohort identified significantly elevated scores in those with BPNETs compared to controls. The majority of controls (80%) had scores < 14% consistent with the absence of disease. In the remainder, none exhibited levels > 20%. This confirms low scored expression (NETest score: 4 ± 5%) in the control group. A sub-analysis of the NET cohort identified that the highest scores were identifiable in those with progressive lung carcinoid disease. All progressive disease patients exhibited scores > 40%. In contrast, > 90% of patients with stable disease had scores < 40%. These data demonstrate that the minimal, low activity and intermediate-high activity ranges established for GEP-NETs are broadly recapitulated in this BPNET cohort. Furthermore, the ability of the score to differentiate progressive/active from stable disease identifies that circulating gene expression measurements accurately correlates with the clinical phenotype. We envisage that cut-offs for the scores may require modification as the test is evaluated either to specifically diagnose BPNETs or predict recurrence after surgery.

To summarize, measurement of neuroendocrine-specific circulating mRNA levels in blood and scoring of this gene expression accurately identified BPNETs. Our evaluation of transcript profiles in tumor tissue and matched blood pairs as well as confirmation via a transcriptome-based assessment and in model cell lines provides scientific support for assessing the role that such a 51 neuroendocrine marker gene panel may play in the clinical management of BPNET. Specificity of the BPNET signature was demonstrated by comparison to benign lung disease and lung cancers. The ability of the signature to distinguish progressive BPNET disease from stable disease and controls suggest this blood-based biomarker tool could function as a clinically informative methodology to facilitate the management of BPNET. We envisage that quantification and scoring of circulating neuroendocrine-specific gene expression will provide real-time information to aid clinical management of bronchopulmonary carcinoids.

## MATERIALS AND METHODS

### Strategy

**Figure 6:**
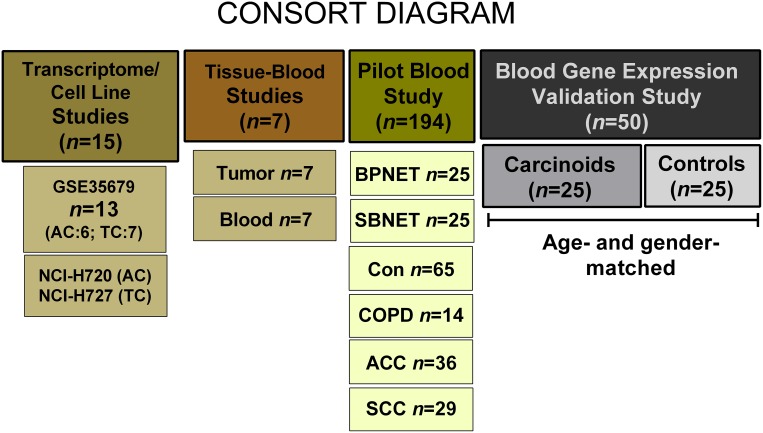
Methodological approach The transcriptome evaluation study included 13 publicly available tumor tissue transcriptomes (Reference 15). The two model cell lines that were examined included the AC-like H720 and the TC-like H727. Gene expression was examined in 7 tumor tissues and time-matched blood samples (patient details in Table 2). The pilot study comprised 194 samples. These included 25 lung and 25 small bowel carcinoids, 65 controls, 14 COPD (benign lung disease), 36 adenocarcinomas and 29 squamous cell carcinomas. The validation study was undertaken in 25 age- and gender-matched BPNET and controls (patient details in Table 3). ACC = adenocarcinoma; Con = Controls; COPD = chronic obstructive pulmonary disease; BPNET = bronchopulmonary neuroendocrine tumors; SBNET = small bowel neuroendocrine tumors; SCC = squamous cell carcinoma.

To evaluate whether a neuroendocrine tumor-specific gene expression assay for gastrointestinal tumors was applicable to BPNET, we initially examined whether mRNA for the 51 gut neuroendocrine marker genes were detected in published BPNET transcriptomes (TC/AC) (Figure 6). We then evaluated whether these genes were amplified in two model bronchopulmonary NCI-NET cell lines and then in seven surgically resected BPNETs (Table 2). Thereafter, we examined expression in blood samples in these surgical patients to evaluate the correlation between tissue and circulating levels. We then undertook a pilot study in 194 samples including controls (*n* = 65), benign lung diseases (*n* = 14), BPNET (*n* = 25), small bowel (SB) NET (*n* = 25) and lung cancers: adenocarcinomas (*n* = 36) and squamous cell carcinomas (*n* = 29) to examine whether 51 marker genes were detected in blood and if expression levels were comparable in BPNETs and small bowel tumors. To validate the assay, we examined the gene expression score in an independent set of age- and gender-matched BPNETs and controls (Table 3). In this study, we evaluated whether scored expression levels (NETest score: ranging 0–100%) firstly differentiated tumor from control and secondly, distinguished progressive from stable disease.

**Table 2:** Seven matched tumor tissue-blood samples

Surgical Tissue-Blood Matched Samples					
Sample	Gender	Age	Type	Treatment	Status
N1	M	68	TC	None	PD
N2	F	37	TC	None	PD
N3	M	46	AC^*^	None	PD
N4	M	49	AC^*^	None	PD
N5	F	59	AC	None	PD
N6	F	68	AC	None	PD
N7	F	70	AC^*^	Sandostatin	PD

AC = atypical carcinoid; PD = progressive disease; TC = typical carcinoid

^*^Metastases.

**Table 3:** Demographics–validation set

BPNETs (*n* = 25)						Controls (*n* = 25)			
Sample	Gender	Age	Type	Treatment	Status	Sample	Gender	Age	Pathology
N1	M	46	TC	None	SD	C1	M	45	None
N2	F	46	TC^*^	None	SD	C2	F	43	IBD
N3	F	48	AC	None	PD	C3	F	46	None
N4	M	53	AC^*^	Captem	PD	C4	M	50	GERD
N5	F	53	TC^*^	Sandostatin	PD	C5	F	51	IBD
N6	F	58	TC^*^	Sandostatin	SD	C6	F	58	None
N7	F	58	TC	None	SD	C7	F	58	None
N8	F	59	TC	None	SD	C8	F	60	GERD
N9	F	61	AC	None	PD	C9	F	60	Cyst
N10	F	61	TC^*^	None	PD	C10	F	61	Cyst
N11	F	62	TC	None	PD	C11	F	62	IBD
N12	F	63	TC	None	SD	C12	F	64	None
N13	F	63	TC	None	SD	C13	F	64	None
N14	F	63	TC	None	PD	C14	F	65	None
N15	F	65	TC	None	SD	C15	F	65	Cyst
N16	F	65	AC	None	PD	C16	F	66	GERD
N17	F	65	TC	Sandostatin	PD	C17	F	67	None
N18	M	66	TC^*^	Sandostatin	SD	C18	M	67	GERD
N19	F	68	TC	None	SD	C19	F	67	Cyst
N20	F	68	TC	None	SD	C20	F	68	None
N21	M	69	AC	None	SD	C21	M	70	None
N22	F	70	AC	Sandostatin	PD	C22	F	71	None
N23	F	74	TC	None	SD	C23	F	72	GERD
N24	F	75	TC	None	PD	C24	F	74	None
N25	F	77	AC^*^	None	PD	C25	F	75	Cyst

AC = atypical carcinoid; cyst = benign pancreatic cyst; GERD = gastroesophageal disease; IBD = inflammatory bowel disease; PD = progressive disease; SD = stable disease; TC = typical carcinoid

^*^Metastatic disease.

### Patients and samples

All provided informed consent authorized by local ethics committees. Whole blood (10 ml) for transcript analysis was collected either immediately prior to surgery (surgical cohort) or at regular follow-up (BPNET) or controls. Tumor tissue was evaluated following histopathological verification of disease. Tumor samples and macroscopically normal tissue were snap-frozen and stored at −80°C until analysis. Anatomical imaging (CT/MRI) was used for staging to evaluate progression (RECIST 1.0 criteria).

### Matched tumor tissue-blood pairs

This included 7 BPNETs (Table 2). All were progressive at the time of surgery, the majority (*n* = 5, 71%) were identified with atypical carcinoid histology. Metastases were present in 3 (43%). One of the patients was being treated at the time of surgery (Sandostatin 30 mg).

### Pilot study set

Gene expression data from previously published data including controls (*n* = 65), benign lung diseases (*n* = 14), BPNET (*n* = 25), small bowel (SB) NET (*n* = 25) and lung cancers: adenocarcinomas (*n* = 36) and squamous cell carcinomas (*n* = 29) was evaluated [17, 22, 24, 44, 45].

### Validation set

This included BPNET patients and controls, matching the 25 cases with a control (1:1) by sex and age to within 3 years. The ethnicity was exclusively Caucasian. The demographics of each group are included in Table 3. There were no differences in sex distribution (M:F 4:21, both groups) or age between the two groups (BPNETs: mean 62.2 years, range: 46–77; controls: mean 61.9 years, range: 43–75). The majority of NETs were TC (*n* = 18, 72%). Seven (28%) had metastases, 6 (24%) were undergoing treatment (5 Sandostatin, 1 CapTem), and 13 (52%) were RECIST stable at blood draw. The control group was included from patients undergoing upper endoscopy for GI-related complaints. Twelve (48%) had no macroscopic disease, 5 (20%) benign pancreatic cysts (confirmed by pathology), 5 (20%) had GERD, and 3 (12%) IBD.

### Transcriptome analysis (*n* = 13)

Thirteen BPNET samples (TC: 6; AC: 7 publicly available Gene Expression Omnibus [GEO] accession number GSE35679) [15] were evaluated using TAC 3.0 software. U133 Plus 2.0 Arrays were normalized using Robust Multi-Array Average (RMA) and log_2_ bi-weighted expression generated. Probes present in > 50% of samples were retained. Hierarchical clustering (1-Pearson correlation) included complete linkage. Individual NET gene analysis using log_2_ values was performed for the two groups using Morpheus (https://software.broadinstitute.org/morpheus/).

### Cell line transcript analysis (*n* = 2)

NCI-H720 [H720] (ATCC^®^ CRL-5838^™^), an AC-like cell line, del(p14-p23), t(3p;4p) and NCI-H727 [H727] (ATCC^®^ CRL-5815^™^), a TC-like cell line, were evaluated [46]. RNA was extracted from logarithmic-growing cells (TRIzol^®^, Invitrogen, USA) [25] and real-time PCR analysis performed using Assays-on-Demand products and the ABI7900 Sequence Detection System [25]. Data was normalized using *ALG9* and ΔΔC_T_ [25].

### Analysis of matched tissue-blood sample pairs (*n* = 7)

Seven NET tumors (AC: *n* = 5; TC: *n* = 2–Table 2) with matched whole blood collected immediately prior to surgery. RNA was extracted from tumor tissue as described (TRIzol^®^, Invitrogen, USA) [25] and qPCR analysis performed. The data was normalized (*ALG9*, ΔΔC_T_) [25]. For fold change analysis, tumor gene expression was compared to normalized values in normal parenchyma tissue collected at the same time. Matched blood samples were processed as described below. For fold-change analysis, gene expression in the 7 BPNET bloods was compared to known expression of the 51 marker genes in 90 previously evaluated healthy controls [17, 22, 24, 44].

### Blood-based transcript measurement

Details of PCR methodology, mathematical analysis and validation have been published in detail [17, 22, 24, 44] comprising a 2-step protocol (RNA isolation, cDNA production and qPCR) [24, 44] from EDTA-collected whole blood [24, 44]. Target transcript levels are normalized and quantified versus a known population control [24]. Thereafter, multianalyte algorithm analyses (MAAA) are undertaken (*Support Vector Machine*, *Linear Discriminant Analysis*, *K-Nearest Neighbor* and *Naive Bayes Classifier*) for categorization into two different groups (tumor or “not a tumor”) using “majority vote” [24]. Final results are expressed as an activity index from 0–100% [17], based on the integration of the majority vote and summated expression of gene expression including regulation of proliferation, epigenetic regulation, growth factor signaling and pluripotency [17]. The activity index ranges that conform to a clinical disease assessment (imagery and clinical status) are: minimal activity: < 0–14%, low activity: 14–40%, moderate: 41–79%, and high activity: 80–100% [17, 22, 23, 47]. The upper limit of normal is 14%.

### Statistical analysis

These included regression analysis (Pearson and Spearman: tumor-blood pair correlations following log-transformation of normalized expression levels; or fold change compared to tumor tissue or blood; and separately, BPNET-SBNET gene expression correlations), Fisher's (2-tailed), and non-parametric (Mann-Whitney 2-tailed) measurements. PCA and hierarchical clustering of normalized gene expression data (non-transformed) was undertaken using Morpheus software (https://software.broadinstitute.org/morpheus/). Prism 6.0 for Windows (GraphPad Software, La Jolla California USA, www.graphpad.com) and MedCalc Statistical Software version 16.2.1 (MedCalc Software bvba, Ostend, Belgium; http://www.medcalc.org; 2017) were utilized. *P* < 0.05 was considered significant.

## Abbreviations

AC: atypical carcinoid
ACC: adenocarcinoma
ATCC: American Type Culture Collection
C_T_: cycle time
BP: bronchopulmonary
CgA: chromogranin A
COPD: chronic obstructive pulmonary disease
DBP: vitamin D binding protein
EDTA: Ethylenediaminetetraacetic acid
EGFR: EGF receptor
GEP: gastro-entero-pancreatic
NET: neuroendocrine tumor
PCR: polymerase chain reaction
RECIST: Response Evaluation Criteria in Solid Tumors
SBNET: small bowel neuroendocrine tumor
SCC: squamous cell carcinoma
TC: typical carcinoid
